# Emphysematous epididymo-orchitis as a camouflage of prostate invasion secondary to rectum cancer: A case report

**DOI:** 10.1097/MD.0000000000004385

**Published:** 2016-07-29

**Authors:** Ching-Heng Yen, Chin-Yu Liu, Tai-Lung Cha, Sheng-Tang Wu, En Meng, Guang-Huan Sun, Dah-Shyong Yu, Hong-I Chen, Sun-Yran Chang, Chih-Wei Tsao

**Affiliations:** a Division of Urology, Department of Surgery, Tri-Service General Hospital, National Defense Medical Center; b Division of Urology, Department of Surgery, Tri-Service General Hospital, Song-Shan Branch, Taipei; c Department of Nutritional Science, Fu Jen Catholic University, New Taipei City; d Graduate Institute of Medical Sciences, Chang Jung Christian University, Tainan, Taiwan.

**Keywords:** epididymo-orchitis, prostate gland, rectum cancer

## Abstract

**Introduction:**

Emphysematous epididymo-orchitis is a rare cause of acute scrotum pain characterized by gas formation within the tissue. Diabetes mellitus and recto-seminal fistula secondary to sigmoid diverticulitis are generally accepted as being responsible for this disease. However, prostate invasion secondary to rectal cancer may be considered to be a newly identified pathogenetic mechanism. Herein, we report this rare case and illustrate the pathogenesis.

**Case presentation:**

A 69-year-old man arrived at our emergency department presenting with sepsis and acute scrotal pain. Emphysematous epididymo-orchitis was diagnosed by scrotal sonography initially; however, advanced rectal cancer with prostate invasion was diagnosed by CT after a recurrent episode. An exploratory laparotomy with abdominoperineal resection and radical prostectomy were performed after neoadjuvant chemoradiotherapy. Histopathologic analysis confirmed the previous diagnosis. Emphysematous epididymo-orchitis caused by advanced rectal cancer is very rare, and our case is the first to be reported according to a literature search. Neoadjuvant chemoradiotherapy plus extended surgery can achieve a good oncological outcome.

**Conclusion:**

This case indicated that the very rare presentation as emphysematous epididymo-orchitis caused by locally advanced colorectal cancer.

## 1 Introduction

Acute epididymo-orchitis is an inflammation of the epididymis, often involving the testis, caused by bacterial infection. Extension of infection from the urethra or bladder is a well-known pathogenesis of acute epididymo-orchitis. Rarely, emphysematous epididymo-orchitis may be characterized by the presence of air within the tissue, presenting a rare cause of acute scrotum pain.^[1]^ Seminal vesicle fistula directly related to colonic diverticulitis^[2]^ and diabetes in patients concomitant with urinary tract infection^[3]^ or testicular artery pseudoaneurysm^[4]^ have been reported as possible pathogenesis pathways. However, the true pathogenesis is unclear owing to the small number of cases. Herein, we describe a case of emphysematous epididymo-orchitis with initial treatment failure. Computed tomography (CT) of the pelvis demonstrated a rectal mass, which was found to have directly invaded into the prostate after further evaluation. Pathologic analysis revealed adenocarcinoma of the rectum with prostate invasion. We assumed that anatomic change of the invasive tumor induced microfistula formation between the rectum and prostate. Furthermore, intestinal bacteria induced prostate abscess formation and extended from the ejaculatory duct to the epididymis. Finally, the bacteria with gas production resulted in the clinical emphysematous epididymo-orchitis. Interference in prostatic intraductal drainage by the bulging rectal tumor also exacerbated the bacterial retrograde infection. This hypothesis provides a more complete explanation of the pathogenesis of emphysematous epididymo-orchitis.

## 2 Case presentation

**Figure 1:**
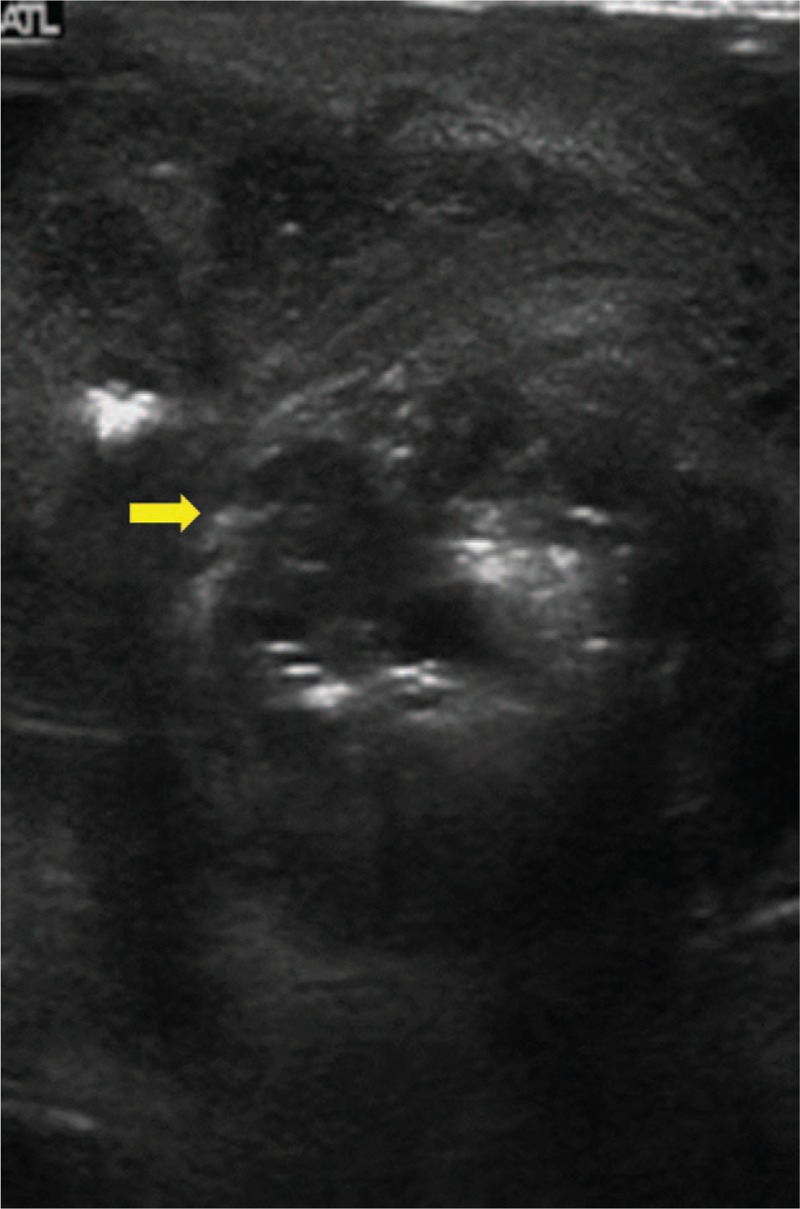
Scrotum sonography showed bright spots and hypoechoic areas.

**Figure 2:**
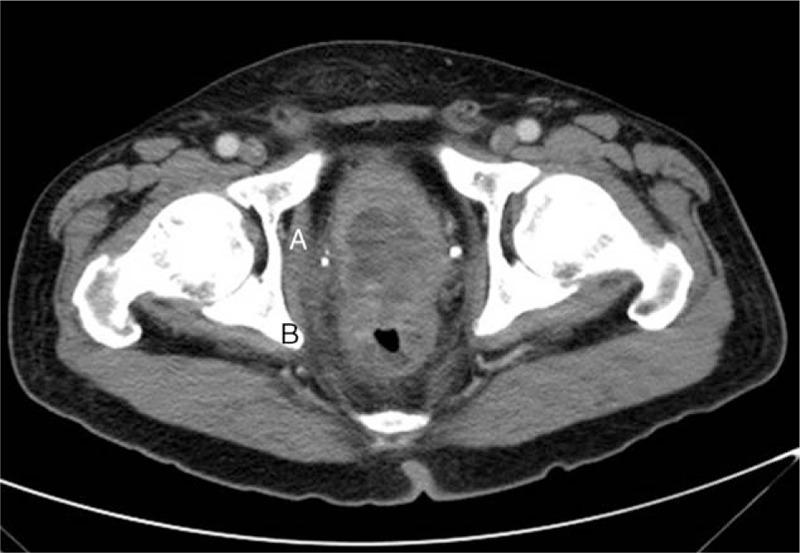
Computed tomography (CT) showed (A) a heterogeneous density of the prostate with central low attenuation, and (B) thickening of the rectum wall.

**Figure 3:**
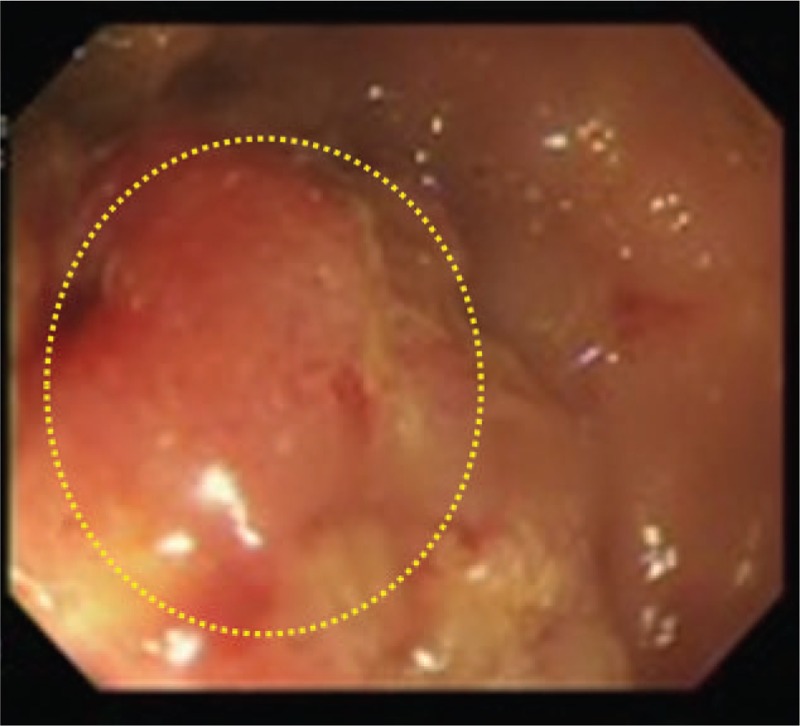
Colonoscopy showed a tumor lesion 5 cm above the anal verge.

**Figure 4:**
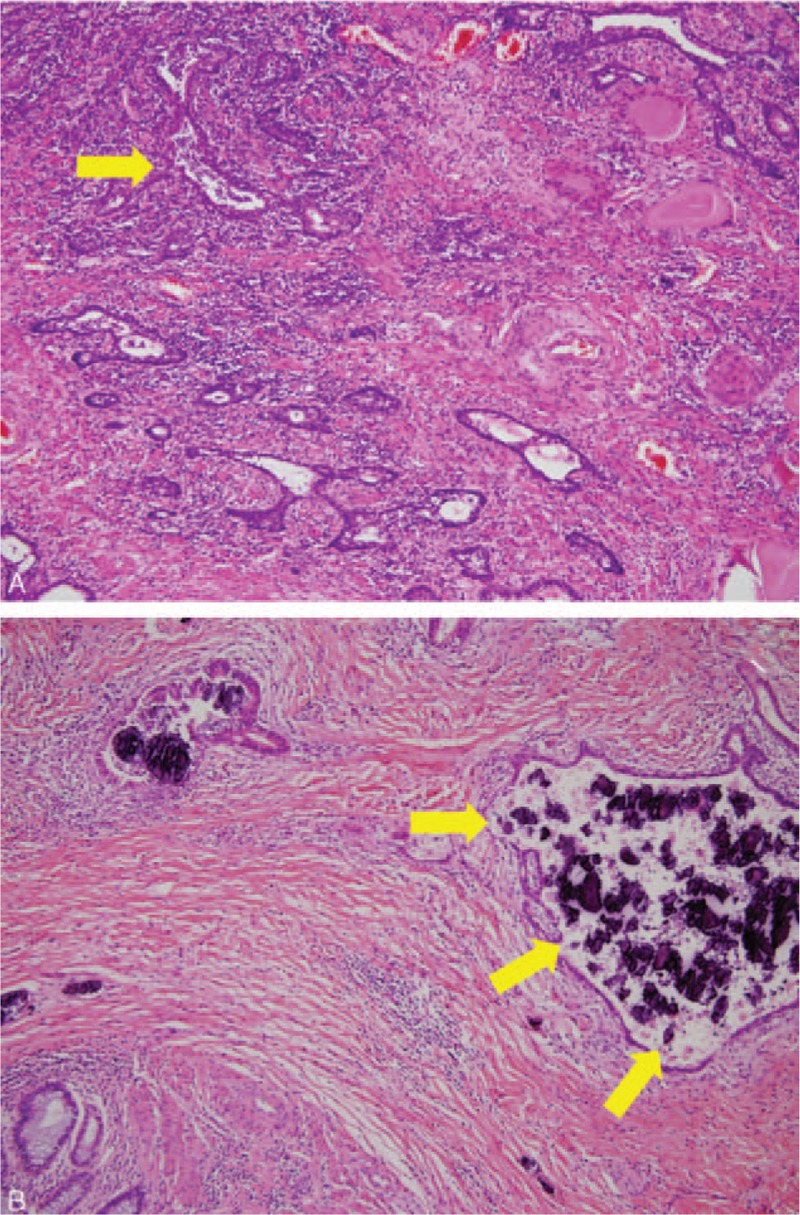
(A) The hematoxylin and eosin (H&E) stain section showed glandular tumor cells infiltrating into the stroma of the colon (×100). (B) The H&E section showed tumor cells arranged in a glandular structure associated with many inflammatory cells infiltrating into the prostatic tissue (×100).

We present the case of a 69-year-old male who arrived at the Emergency Department with right side acute scrotal pain. No history of trauma, recent sexual exposure, or major medical disease was documented, with only the symptom of intermittent diarrhea in recent months being reported. Vital signs were as follows: body temperature of 38°C, pulse rate of 72 per minute, respiration rate of 19 per minute, blood pressure of 76/54 mm Hg. Physical examination revealed that the pain was located on the right side scrotum and it extended to the ipsilateral inguinal region without perineum involved. The patient's hemoglobin level was 12.7 g/dL, the white blood cell count was 12,240 /μL, and the C reactive protein (CRP) level was 14.55 mg/dL. Scrotum sonography demonstrated some bright spots and hypoechoic areas (Fig. 1). Acute epididymo-orchitis with abscess and gas formation was impressed. The patient underwent right unilateral orchiectomy and debridement. A pus culture showed *Bacteroides fragilis* and *Clostridium* spp. The patient was discharged after adequate antibiotics with ceftriaxone and metronidazole at stable wound condition. However, recurrent right side scrotal pain was noted with purulent discharge from the previous surgical wound after 1 month, which pathogen proved as mixed organisms including *B fragilis*, *Clostridium* spp., and *Klebsiella pneumonia*. Digital rectum examination demonstrated moderate enlarged prostate with induration, without palpable rectal mass. Due to inexplicable clinical features, CT of the pelvis and scrotum was performed, which demonstrated a heterogeneous density of the prostate with central low attenuation, whole wall-thickening of the rectum, and enlargement of lymph nodes (Fig. 2). Advanced-stage rectal cancer with prostate abscess was assumed. The colonoscopy showed a tumor lesion with annular type 5 cm above the anal verge (Fig. 3), then a biopsy was performed and the pathologic analysis confirmed adenocarcinoma of the rectum. The immediate tumor markers showed carcinoembryonic antigen (CEA) of 9.40 ng/mL and carbohydrate antigen 19-9 (CA19-9) of 21.30 U/mL. The positron emission tomography (PET) scan revealed no abnormal fluorodeoxyglucose (FDG) uptake throughout whole body region and the presumed case clinical stage of rectum adenocarcinoma was T4N1M0. The suprapubic cystostomy for urinary diversion and T-loop colostomy were performed for infection control. Subsequently, the patient received neoadjuvant chemoradiotherapy with high dosage 5-fluorouracil and leucovorin. Finally, he underwent an exploratory laparotomy with abdominoperineal resection and radical prostatectomy. Pathologic analysis revealed moderately differentiated adenocarcinoma of colonic origin with direct invasion into the bilateral prostatic tissue (Fig. 4). The final stage was ypT4bN1bM0. The patient received adjuvant chemotherapy with oral form capecitabine until the present time. The recent tumor marker results after resection of rectum tumor were a CEA level of 1.16 ng/mL and a CA19-9 value of 11.36 U/mL. No evidence of tumor recurrence was found by imaging study.

Written informed consent to publish the case report was provided by the patient, and the consent procedure was approved by the Ethics Committee of Tri-Service General Hospital.

## 3 Discussion

Emphysematous epididymo-orchitis is a very rare disease, and only 3 cases have been reported.^[1–3]^ Acute scrotal pain, swelling of the scrotum, and fever are most common symptoms. Imaging findings of ultrasound and CT have demonstrated air within the testis to be the main characteristic in the few reported cases.^[1–3]^ Unlike extended infection, which is a well-known mechanism of acute epididymitis, the pathogenesis of emphysematous epididymo-orchitis remains unclear. Diabetes mellitus^[3]^ and recto-seminal fistula secondary to sigmoid diverticulitis^[2]^ have been reported to be pathogenetic mechanisms. However, only 1 colorectal cancer patient has been reported to be related to the epididymo-orchitis in the literature.^[5]^

Advanced colorectal cancer invading the urinary tract is not uncommon, and owing to the close anatomic relationship, approximately 5% of primary colorectal cancers are locally advanced to the urinary system.^[6]^ Urinary tract involvement in cases of colorectal cancer is by means of direct invasion or fistula formation.^[6–8]^ In addition, colon cancer can induce Fournier gangrene.^[9,10]^ Therefore, the urinary tract can be affected by direct tumor invasion or tumor-associated inflammatory processes.

**Figure 5:**
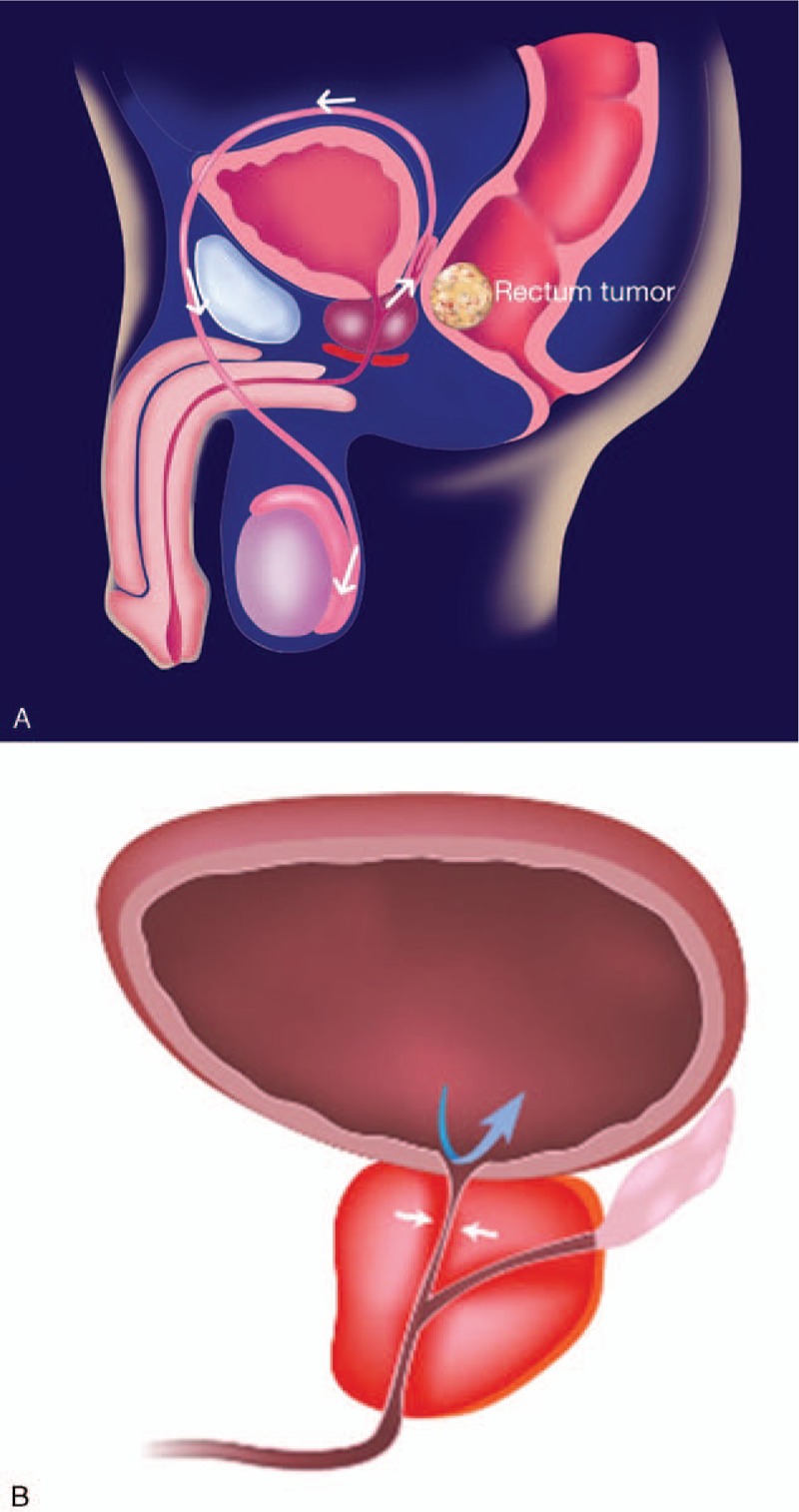
Illustration of the pathogenesis of emphysematous epididymo-orchitis. (A) A colorectal tumor-associated inflammatory process induced a fistula, followed by prostate abscess formation and ascending bacterial infection via the verumontanum to the epidydimis. (B) Inflamed prostate with poor prostatic intraductal drainage and backflow of urine.

Emphysematous epididymo-orchitis can be an unusual presentation of sigmoid diverticulitis.^[2]^ Colo-urinary tract fistula complicating colonic diverticulitis has been reported. A fistula between the colon and urinary tract is most common among other types, and urinary tract infection is the main symptom.^[11]^ Colorectal cancer also can induce the same inflammatory process and fistula formation. Bruketa et al^[9]^ ever concluded that the perforated rectal cancer could induce Fournier gangrene with perineum fascia extension and absence of testis involvement. However our case presented as annular rectal cancer with prostate invasion, which hypothesized to process the development of emphysematous epididymo-orchitis through the vas deferens via backflow of gas-producing bacteria. With the observation that the emphysematous epididymo-orchitis without perineum fascia extension was noted in our case, we postulated that there existed a fistula between rectum and prostate. Further, intestinal bacteria induced prostate abscess and infection extending from the ejaculatory duct to the epididymis. The pathogenesis of our presented case is quite different from perforated rectal cancer inducing Fournier gangrene. In our case, the abscess and infection were evidenced by recurrent infection and bacterial culture analysis. Moreover the inflamed prostate obstructed the prostatic urethra, causing poor prostatic intraductal drainage and backflow of urine. This is also an aggravating factor in emphysematous epididymo-orchitis. In conclusion, the hypothesized pathogenesis was illustrated in the schematic diagram (Fig. 5).

Total pelvic exenteration is the gold-standard treatment for locally advanced colorectal cancer^[12–16]^; however, a bladder-sparing procedure allows conservative surgical treatment to be administered in selected patients.^[17]^ In addition, neoadjuvant chemoradiotherapy with capecitabine before total pelvic exenteration is effective for the treatment of locally advanced sigmoid colon cancer.^[18]^ In a recent review article,^[19]^ neoadjuvant chemoradiotherapy was reported to result in a higher R0 rate than surgery alone, and a longer overall survival of 85% to 75% within 3 to 5 years was achieved. In our case, we performed suprapubic cystostomy for urinary diversion and T-loop colostomy for infection control first. The patient subsequently received neoadjuvant chemoradiotherapy and an exploratory laparotomy with abdominoperineal resection. Postoperative adjuvant chemotherapy is currently in progress. No distant metastasis or recurrence has been observed. Therefore, multimodal therapy and extended surgery result in a good prognosis in patients with locally advanced colorectal cancer.

## 4 Conclusion

Emphysematous epididymo-orchitis caused by locally advanced colorectal cancer is very rare, and our case was the first to be reported according to a search of the literature. A fistula between the urinary tract and colon in addition to an extended bacterial infection is the most reasonable pathogenetic mechanism of emphysematous epididymo-orchitis. Neoadjuvant chemoradiotherapy with total pelvic exenteration can achieve a successful oncological outcome.
